# Supplementary material for: The epidemiological impact of the Canadian COVID Alert app
Source: Can J Public Health. 2022 Jun 7;113(4):519–27. doi: 10.17269/s41997-022-00632-w (PMC9172601; doi:10.17269/s41997-022-00632-w)
Supplement: Supplementary file 1 — (DOCX 49.5 kb) [file 41997_2022_632_MOESM1_ESM.docx]

**Supplementary Appendix**

**The epidemiological impact of the Canadian COVID Alert App**

In this Appendix, we provide detailed definitions of the terms needed to model the impact of the exposure notification app on averted cases due to COVID-19.

**The number of notifications received**

The process to obtain a one-time key to upload a positive COVID-19 test is different in each province and territory, thus it is possible that some users do not ever receive a key. Further, COVID-19 positive declaration is not mandatory and users who test positive have only 24 hours to enter the key in the app. Therefore, the number of notifications received during the study period is an underestimate of the number of users who test positive.

**Secondary attack rate**

The secondary attack rate (SAR) is defined as the probability that an infection occurs among individuals notified by the COVID Alert app within a reasonable incubation period (e.g., 14 days). It can provide an indication of how social interactions relate to transmission risk. The SAR in Wymant et al. (2021) is 6.02% (CI: 5.96 - 6.09); the lower bound of SAR estimate in Segal et al. (2021) is 5.1%. We therefore consider two plausible SAR values: 5% and 6%.

**The generation time distribution**

The generation time (or generation interval) is defined for source-recipient transmission pairs as the interval between the time of infection of the index case and the time of infection of the secondary case. It is typically difficult to estimate this quantity directly unless the interval of exposure is short for both the source case and the recipient case. Instead, the generation time is usually estimated indirectly from intervals of exposure and onset of symptoms. Ferretti et al. (2020) directly estimate the generation time distribution from 191 source-recipient pairs, with known time of onset of symptoms, intervals of exposure, and a meta-distribution of incubation period. The distribution is described by a Weibull distribution (shape=3.2862 and scale=6.1244) with mean generation time equal to 5.5 days (Ferretti et al. 2020b).

**The expected fraction of transmissions prevented by strict quarantine**

The expected fraction of transmission preventable if an infectious individual strictly adheres to quarantine after receiving a notification depends on the delay time, i.e., the interval (delay) between exposure and exposure notification. We adopt the estimated mean time of 5.46 days from Segal et al. (2021). The expected fraction of transmissions prevented by receipt of exposure notification is the upper tail probability of the generation time distribution (i.e., P(X ≥ 5.46)), which corresponds to approximately 0.50.

**The quarantine effectiveness**

Adherence to quarantine is critical but difficult to access reliably. The plausible values, 45% and 65%, are considered based on estimations in Wymant et al. (2021) and Segal et al. (2021) in which effectiveness of quarantine is estimated based on surveys and assumptions. The overall effectiveness of quarantine estimation is 61% (with range from 53.25% to 68.74%) in Wymant et al. (2021), and the estimate in Segal et al. (2021) is 53%.

**The expected size of the full transmission chain**

The size of the transmission chain is a function of the number of cases during the study period. Let $d\left( x,t \right)$ denote the number of diagnosed cases among the whole population in province $x$ at time $t$. The number of cases averted at time $T$ (i.e., the size of the whole chain caused by a single transmission at time $t$), is $\frac{1}{t_{g}}\exp\left[ \int_{t}^{T} r_{t^{'}}\left( x \right)dt^{'} \right]$ for any $t<T$, where $t_{g}$ is the mean generation time and $r_{t}\left( x \right)$ is the epidemic growth rate at time $t$. Here we follow the assumptions of Wymant et al., 2021; specifically, it is assumed that local epidemics do not mix and that the extra cases do not affect the epidemic dynamic (i.e., the underlying growth rate $r_{t}\left( x \right)$ does not change with the additional cases). Then we obtain $d\left( x,T \right)=d\left( x,t \right)\exp\left[ \int_{t}^{T} r_{t^{'}}\left( x \right)dt^{'} \right]$ for any $t<T$. Therefore, instead of estimating the growth rate $r_{t}\left( x \right)$, we can use the factor $\frac{d\left( x,T \right)}{d\left( x,t \right)t_{g}}$ to obtain the number of cases averted at time $T$ for each transmission time $t$. The number of cases $d\left( x,t \right)$ is estimated by taking the weekly moving average of new daily cases in each Canadian province.

**Supplementary Figure 1.** Estimated daily COVID-19 cases averted by province over time

**References**

Ferretti, Luca, Alice Ledda, Chris Wymant, Lele Zhao, Virginia Ledda, Lucie Abeler-Dörner, Michelle Kendall et al. (2020). The timing of COVID-19 transmission. *medRxiv*; published online Sept 7. https://doi.org/10.1101/2020.09.04.20188516 (preprint).

Segal, C., Zhang, Z., Karras, B. T., Revere, D., Zane, G., and Baseman, J. G. (2021). Early epidemiological evidence of public health value of WA Notify, a smartphone-based exposure notification tool: Modeling COVID-19 cases averted in Washington state.

medRxiv. https://www.medrxiv.org/content/10.1101/2021.06.04.21257951v4

Wymant, C., Ferretti, L., Tsallis, D., Charalambides, M., Abeler-Dörner, L., Bonsall, D., Hinch, R., Kendall, M., Milsom, L., Ayres, M., Holmes, C., Briers, M., and Fraser, C. (2021). The epidemiological impact of the NHS COVID-19 App. *Nature*, 594(7863), 408-412.
